# Stabilizing Macular Edema Fluctuations: Outcomes of Intravitreal Fluocinolone Acetonide for Diabetic Macular Edema and Non-Infectious Uveitis

**DOI:** 10.3390/jcm14082849

**Published:** 2025-04-21

**Authors:** Bettina Hohberger, Melanie Royer, Cindy Sheree Flamann, Antonio Bergua

**Affiliations:** 1 Department of Ophthalmology, Uniklinikum Erlangen, Friedrich-Alexander-University Erlangen-Nürnberg, 91054 Erlangen, Germany; bettina.hohberger@uk-erlangen.de; 2 Alimera Sciences Ophthalmologie GmbH, 10709 Berlin, Germany

**Keywords:** fluocinolone acetonide, diabetic macular edema, non-infectious uveitis, uveitis, retinal fluctuations, Iluvien

## Abstract

**Background/Objectives**: Chronic macular edema (CME) is a common complication of diabetic retinopathy or non-infectious uveitis affecting the posterior segment (NIU-PS). Alongside anti-VEGF therapy, glucocorticoids are frequently used to manage CME. Given the heterogeneous nature of patients’ medical history, their social conditions, and disease manifestations, individualized treatment is essential for optimal outcomes. This study assesses the effectiveness of intravitreal fluocinolone acetonide (FA) (Iluvien^®^) in treating persistent and recurrent macular edema in clinical practice at the University Hospital of Erlangen–Nuremberg, Germany. **Methods**: A total of 46 eyes with diabetic macular edema (DME) (21 eyes) and NIU-PS (25 eyes) were retrospectively analyzed over a follow-up period of up to 36 months. Since persistent retinal thickness fluctuations are linked to long-term retinal damage and functional decline, this study analyzed central retinal thickness (CRT)—including its fluctuations measured as CRT amplitude—alongside BCVA as the primary outcomes. **Results**: After an initial decrease in CRT in the first year after FA treatment, the maximum CRT amplitude significantly decreased in the following years. For patients with DME, CRT amplitude reduced from 271.4 µm to 91.57 µm in the first year (*p* = 0.0056) and 106.0 µm in the second year (*p* = 0.0109). For patients with NIU-PS, CRT amplitude decreased from 185.2 µm to 87.7 µm in the first year (*p* = 0.0131) and 97.3 µm in the second year (*p* = 0.0375). Mean BCVA remained stable in both cohorts. **Conclusions**: Intravitreal FA proved to be effective in reducing and stabilizing CRT in patients with chronic DME and NIU-PS without losing visual acuity, reducing treatment burden.

## 1. Introduction

Even with today’s medical and technical possibilities to detect and monitor diabetes mellitus, it remains a major global public health problem—including its comorbidities like diabetic macular edema (DME). By 2045, diabetes cases are projected to rise from 537 million in 2021 to 783 million worldwide [1], and also, in Germany, patient rates are high with an estimated total number of 8 million people diagnosed [2]. DME incidence is expected to increase in line with increasing diabetes cases [3]. DME pathogenesis involves elevated vascular endothelial growth factor (VEGF) levels, along with rising inflammatory mediators like cytokines during DME progression and duration, highlighting the role of inflammation in its progression [4,5].

In uveitis, the role of inflammatory processes is even more prominent. This chronic disease affects the uveal tissue and is the fifth or sixth leading cause of blindness in Western countries, accounting for 10–15% of total blindness cases [6,7]. Hence, preventing inflammatory episodes and recurrences is critical for maintaining visual acuity (VA) and quality of life, particularly as young adults are often affected [8,9]. Both retinopathies impose a significant treatment burden on patients, involving time-consuming appointments, psychological stress, and the need for effective communication and adherence, while healthcare systems are overwhelmed, resulting in long waiting times and less personalized care [10,11,12].

Significant retinal thickness fluctuations present a major challenge in the management of DME, often leading to suboptimal clinical outcomes. Prolonged mechanical stress on the retina may cause irreversible structural damage, ultimately contributing to functional vision loss [13,14,15,16,17]. Therefore, long-term inflammation control with stable retinal thickness is essential for preserving retinal function, VA, and quality of life. Steroid treatments, such as the fluocinolone acetonide (FA) implant, provide a stable approach. The long-lasting steroidal 0.19 mg FA implant (Iluvien^®^, Alimera Sciences, Hampshire, UK) constantly releases a microdose of 0.2 µg/day for up to 36 months [18]. In Europe, the FA implant is indicated for the treatment of (i) chronic DME, when other treatment options are not sufficient and successful; and for (ii) the prevention of relapse in eyes with non-infectious uveitis affecting the posterior segment (NIU-PS).

The constant microdosing of FA has been shown to downregulate inflammatory mediators involved in DME pathogenesis and uveitis [5,19]. Numerous clinical trials and real-world studies report reductions in central retinal thickness (CRT) and inflammatory activity, along with stabilization or improvement in VA [20,21,22,23,24,25,26,27,28]. Furthermore, retrospective and prospective studies have indicated that FA therapy is associated with reduced CRT variability [15,29,30]. The larger-scale PALADIN study confirmed these results, demonstrating that the FA implant significantly reduces retinal thickness fluctuations, resulting in improved visual outcomes, particularly in patients with lower baseline CRT variability [31,32].

To assess FA administration in real-world clinical practice at the University Hospital of Erlangen–Nuremberg, in Erlangen, Germany, we retrospectively analyzed a cohort of patients with either DME or NIU-PS that were treated with FA. At this tertiary center, FA is primarily used as a second- or third-line treatment for a highly heterogeneous and severely affected patient cohort. In this study, we focused on CRT fluctuations and whether VA could be stabilized or improved. Safety aspects, as well as the need for retreatment with FA or additional therapies, were also evaluated.

## 2. Materials and Methods

### 2.1. Study Design

This retrospective study, conducted at the Department of Ophthalmology at the University of Erlangen–Nuremberg, Friedrich-Alexander University Erlangen–Nuremberg, in Erlangen, Germany, evaluated the real-world effectiveness of the 0.19 mg fluocinolone acetonide intravitreal implant (FA). Two patient cohorts were analyzed: cohort 1 included those with chronic DME, while cohort 2 comprised patients with NIU-PS. Ethical approval was granted by the Institutional Review Board of the Department of Ophthalmology at Friedrich-Alexander University, and the study protocol adhered to the Declaration of Helsinki.

The present analysis was performed on data extracted between September 2013 and April 2022. All patients treated with FA who had a follow-up period of at least six months were included. Treatment decision to switch to the 0.19 FA was based on (1) insufficient efficacy of prior treatments before switching to FA, (2) persistent or recurrent edema, (3) no improvement in VA to previous treatment, and (4) frequent recurrences during the pre-treatment phase—at the physician’s discretion.

### 2.2. Study Outcomes

Baseline demographics and disease characteristics including age, sex, treatment history, lens status, prior systemic treatments, and treatments for intraocular pressure (IOP) were collected if available. The primary study outcomes were best-corrected visual acuity (BCVA) and central retinal thickness (CRT), which measures the mean retinal thickness within the 1-millimeter-diameter circular field surrounding the foveola. CRT was assessed using spectral-domain optical coherence tomography (OCT) (Spectralis^®^, Heidelberg Engineering GmbH, Heidelberg, Germany). Baseline values for BCVA, CRT, and IOP were collected at the last examination prior to FA injection. The maximal CRT amplitude was determined by calculating the absolute difference between the lowest and highest CRT values during a study period. Secondary outcomes included IOP measurement (Canon Full Auto-Tonometer TX-F, Canon, Krefeld, Germany) and FA-associated complications. Complications were defined as increased IOP, need for IOP-lowering topical treatment or surgery, cataract development, or any incidents requiring surgical implant removal. Type and time point of additional treatments (if necessary) were also documented.

### 2.3. Data Analyses

The above measures were collected at the last examination before and at each examination after the FA injection. Due to the retrospective nature of this study, the number and intervals of examinations varied across cohorts. For consistency, data from the most recent examination within each three-month period (referred to as quartiles) were used for analysis and labeled by year and quartile. Data were analyzed up to 36 months, although longer-term data were available for some patients (see patient cases A and B). Data are shown as descriptive statistics, reported as mean ± standard deviation (SD) or standard error mean (SEM), and as percentages of eyes or patients unless stated otherwise. All datasets were checked for normal distribution. In the case of normally distributed data, a mixed-effects analysis was used. The Kruskall–Wallis test was applied for non-normally distributed data. For all comparisons, statistical significance was set at *p* < 0.05. If the parametric or non-parametric test was statistically significant, post hoc analyses were performed (Sidak’s multiple comparison test or Dunn’s multiple comparisons test). No further adjustments were made for multiple comparisons. Statistical tests were performed using GraphPad Prism 8 (GraphPad Software, Boston, MA, USA).

## 3. Results

### 3.1. Baseline Demographics

Baseline characteristics for DME and NIU-PS cohorts are presented in Table 1. The DME cohort included 21 eyes of 18 patients, with a mean follow-up of 792.5 ± 462.5 days. The mean age of patients treated with the FA implant was 66.6 ± 5.9 years, with the majority being pseudophakic (78.3%). None of the eyes had an IOP > 30 mmHg at baseline. All except one eye (*n* = 20) received prior anti-VEGF, steroid, or laser treatment, with 66.7% (*n* = 14) receiving intravitreal steroids. In total, 61.5% of eyes treated with a dexamethasone implant (8 of 13) were switched to FA after up to two dexamethasone implants (Table S1 in File S1).

The NIU-PS cohort included 25 eyes from 18 patients, with a mean follow-up of 977.2 ± 686.5 days. The mean age was 55.0 ± 11.4 years with 64.0% being pseudophakic and 36.0% being phakic at baseline. Most eyes were diagnosed with non-infectious posterior uveitis (84.0%). A total of 8 eyes were diagnosed with Birdshot retinopathy; 17 eyes were treated due to idiopathic uveitis. Overall, 88.0% of eyes had prior dexamethasone implant treatment, and 59.1% were switched to FA after up to two dexamethasone implants (Table S2 in File S1).

### 3.2. Cohort 1: Patients with DME

#### 3.2.1. Central Retinal Thickness

**Figure 1:**
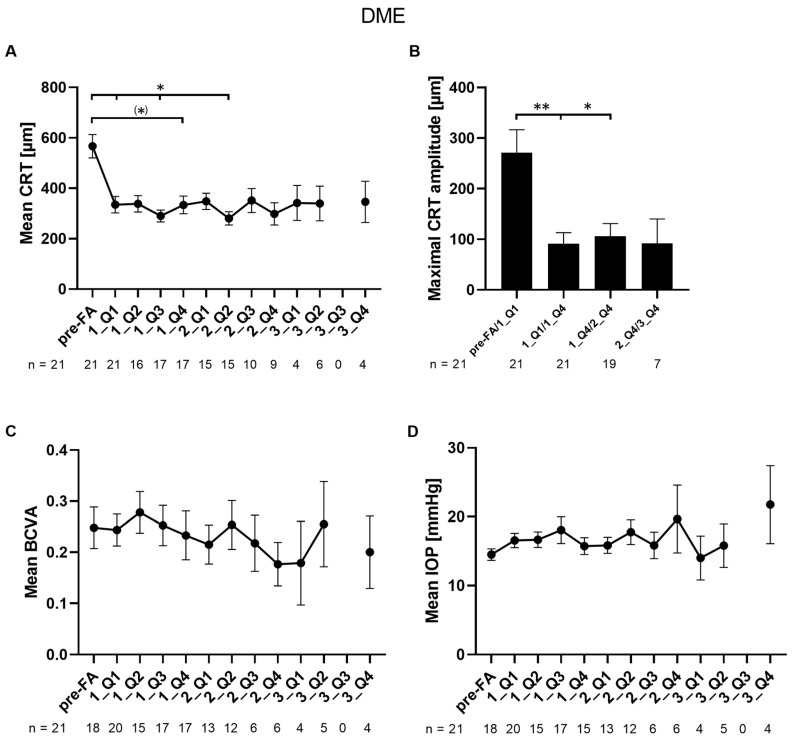
Changes in mean CRT, CRT amplitude, BCVA, and mean IOP in patients with DME: (**A**) mean CRT values are shown per quartile during a follow-up period of three years; (**B**) the absolute value of the maximum CRT amplitude was determined by calculating the difference between the lowest and highest CRT values within a specified period; (**C**) mean BCVA values are shown per quartile during a follow-up period of three years; (**D**) mean IOP values are shown per quartile during a follow-up period of three years. Pre-FA: value pre-FA implantation; x_Qy: quartile y of year x; *n*: number of eyes. Data are shown as mean ± SEM; ** *p* < 0.01; * *p* < 0.05; (*) *p* < 0.06 by trend.

The mean CRT significantly improved after FA during quartiles one and three of the first year and quartile two of the second year compared to baseline (566.7 vs. 335.2, vs. 290.1, and vs. 280.5 µm, respectively; *p* = 0.0284/0.0145/0.0256) and showed a numerical reduction during quartile four of the first year (566.7 vs. 333.8 µm; *p* = 0.0516) (Figure 1A). The minimal mean value of CRT was 280.5 µm, which was observed in quartile two of the second year. All other timepoints did not reach significance. To determine fluctuations in CRT, the maximal CRT amplitudes within certain timeframes were calculated. The maximal CRT amplitude of 271.4 µm ± 206.4 µm was observed within the first quartile of the first year after FA implantation (Figure 1B). The mean maximal CRT amplitudes during the rest of the first year as well as the second year were significantly lower (271.4 vs. 91.57 and vs. 106.0 µm; *p* = 0.0056/0.0109) indicating a stabilization of CRT after FA implantation, leading to less mechanical damage caused by fluctuations.

#### 3.2.2. Visual Acuity

Overall, the BCVA of patients with DME remained stable upon FA implantation during a follow-up time of up to 36 months (Figure 1C). On average, no significant changes were observed.

#### 3.2.3. Safety Outcomes Measures and Cataract-Related Events

Mean IOP remained stable during the follow-up period of up to 36 months (*p* = 0.2113) (Figure 1D). IOP pre-FA treatment was 14.5 ± 3.6 mmHg, non-significantly increasing in quartile four of the first, second, and third year of FA treatment (15.7 ± 4.7 mmHg, 19.7 ± 12.0 mmHg, and 21.8 ± 11.3 mmHg, respectively). In total, three eyes (14.3%) showed an increase of ≥ 10 mmHg at least once during the follow-up period. Four eyes (19%) had an IOD ≥ 30 mmHg during the follow-up period. Details of IOP-related events are shown in Table S3 in File S1. IOP-lowering medication was administered to 42.9% of eyes (*n* = 21) during the follow-up period, which was necessary on average after 289 ± 115 days. Two eyes were treated with IOP-lowering medications pre-FA treatment and continued during the FA follow-up. No IOP-lowering surgeries were necessary during the follow-up.

In total, 23.8% of eyes (5 of a total of 21) were phakic at FA implantation. Two of five phakic eyes (40%) underwent cataract surgery after 335 and 601 days.

### 3.3. Cohort 2: Patients with NIU-PS

#### 3.3.1. Central Retinal Thickness

**Figure 2:**
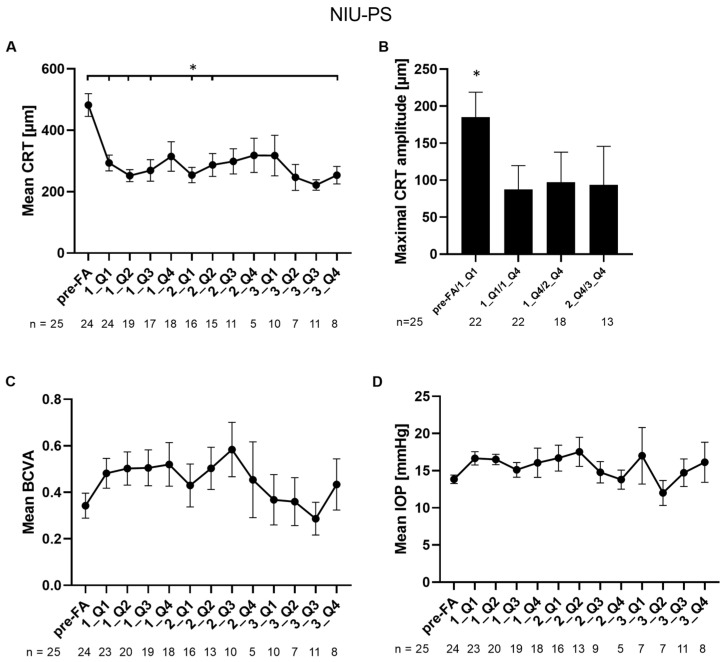
Changes in mean CRT, CRT amplitude, BCVA, and mean IOP in patients with NIU-PS: (**A**) mean CRT values of patients with NIU-PS are shown per quartile during a follow-up period of three years; (**B**) the absolute maximum value of the maximal CRT amplitude of patients with NIU-PS was calculated using the lowest and the highest CRT values within a certain period; (**C**) mean BCVA values are shown per quartile during a follow-up period of three years; (**D**) mean IOP values are shown per quartile during a follow-up period of three years. Pre-FA: value pre-FA implantation; x_Qy: quartile y of year x; *n*: number of eyes. Data are shown as mean ± SEM; * *p* < 0.05.

In eyes with NIU-PS, mean CRT was significantly reduced at quartiles one, two, and three of the first year, quartiles one and two of the second, and quartile four of the third year compared to baseline (482.3 vs. 293.6, 252.2, and 269.1 µm (*p* = 0.000399/0.000840/0.002645), vs. 254.2 and 287.0 µm (*p* = 0.023789/0.011315), and vs. 253.9 µm, respectively; Sidak’s multiple comparisons test *p* < 0.05 *p* = 0.285120) (Figure 2A). The lowest CRT value being significant was found in quartile two of the first year (252.2 ± 85.9 µm). Like in eyes with DME, the maximal CRT amplitude in eyes with NIU-PS was observed during quartile one of the first year with an absolute value of 185.2 ± 157.5 µm (Figure 2B). Maximal CRT amplitudes during the first, second, and third year were significantly lower compared to the first quartile after FA implantation (185.2 vs. 87.7, vs. 97.3 and vs. 93.7 µm; Dunn’s multiple comparisons test; *p* = 0.0131/0.0375/0.0230 <0.05) (Figure 2B).

#### 3.3.2. Visual Acuity

The mean BCVA non-significantly increased until quartile three of the second year compared to the mean BCVA pre-FA implantation (0.34 ± 0.26 BCVA pre-FA treatment vs. 0.58 ± 0.37 BCVA in 1_Q3; Figure 2C). Statistical analysis did not reach significance.

#### 3.3.3. Safety Outcomes Measures and Cataract-Related Events

In eyes with NIU-PS, the mean baseline IOP was 13.8 ± 2.8 mmHg. Mean IOP remained stable and did not significantly change during the follow-up period of up to 36 months (Figure 2D). Six eyes (24%) showed an increase of ≥10 mmHg at least once during the follow-up period. Four eyes (16%) had an IOD ≥ 30 mmHg during the follow-up period. Details of IOP-related events are shown in Table S4 in File S1. IOP-lowering medication was administered to 32.0% of all eyes (*n* = 25), including two eyes that had already been treated before FA implantation. IOP-lowering treatment was started on average after 451 ± 117 days and three eyes underwent IOP-lowering surgeries after 618 ± 429 days. Two of these three eyes were the above-mentioned eyes with IOP-lowering medication pre-FA implantation.

Overall, 36.0% of eyes (9 of a total of 25) were phakic at FA implantation. Six of the originally nine phakic eyes underwent cataract surgery during FA treatment after 357 ± 113 days on average.

#### 3.3.4. Other Adverse Events

In one eye with NIU-PS, vitreous detachment was observed 14 months post-FA implantation. Two eyes with NIU-PS were diagnosed with glaucoma post-FA treatment: one eye was already diagnosed with papillitis and being steroid-responsive to pre-FA treatment; the other eye of a BCR-ABL-positive patient developed secondary glaucoma and was treated with PreserFlo^®^MicroShunt (Santen Pharmaceutical Co. Ltd., Osaka, Japan) 12 months post-FA implantation. Whether these adverse events are directly related to FA treatment cannot be clearly determined. Dislocation of the FA implant was observed in two patients with Birdshot retinopathy: (i) one eye already had a history of dexamethasone implants being dislocated into the anterior chamber. The FA implant was ex- and reimplanted three times; (ii) in the second eye of the same patient, dislocation, and replacement occurred after one month; (iii) in the second patient, the FA implant had to be repositioned after two months. Despite the dislocation, removal, and reimplantation of the FA implants in these above-mentioned patients, no other adverse events like infections or intraocular hypertension leading to removal of the FA implant were reported for all other eyes of both cohorts.

### 3.4. Supplementary Treatments

#### 3.4.1. Cohort 1: Patients with DME

A total of 42.9% of eyes with DME received supplementary treatments within the follow-up period of up to 36 months including laser therapy, intravitreal steroids, anti-VEGF, or a combination of those (for details see Table 2). Supplementary treatment was administered on average 353 ± 328 days post-FA implantation.

#### 3.4.2. Cohort 2: Patients with NIU-PS

In total, 20% of eyes with NIU-PS were treated with supplementary medication, including laser therapy, intravitreal steroids, and anti-VEGF after 332 ± 284 days post-FA implantation (for details see Table 2).

Six of eighteen patients with NIU-PS (44.4%) were additionally treated with systemic therapies at baseline. Two of these six patients additionally received immunosuppressive treatments. During the follow-up period of 36 months, systemic therapy was reduced in two of six patients, and in one patient, systemic treatment was completely stopped.

### 3.5. Reinjections of FA Implants

One eye with DME remained dry at 35 months post-FA implantation (181 µm) and received a second FA implant after 41 months. The eye remained stable in BCVA (0.4) and CRT (<200 µm) for an additional 24 months until the last follow-up visit (see patient A below).

One patient diagnosed with posterior granulomatous uveitis and papillary swelling by sympathetic ophthalmia was preventively reinjected with a second FA implant after 36 months. The macula was, and stayed, dry before and after the second implantation. In this case, FA treatment was in addition to systemic TNF-inhibitory therapy.

The macula of a Birdshot retinopathy patient was dry until 38 months (314 µm) after the first FA implantation. In month 41, an increase in CRT (418 µm) was measured followed by reinjection of a second FA implant in month 44. One month later, CRT was reduced to 167 µm. Forty-seven months after the second FA implant, a third implant was injected, maintaining the macular dryness in this patient (see patient B below).

### 3.6. Case Presentations

#### 3.6.1. Patient A: DME (OS)

An 83-year-old female patient with cystoid DME in her left eye, accompanied by proliferative diabetic retinopathy, had undergone phacoemulsification and intraocular lens implantation in 2009. Her treatment history includes seven bevacizumab intravitreal injections, three triamcinolone injections (last in 2015), and two dexamethasone implants before FA implantation.

**Figure 3:**
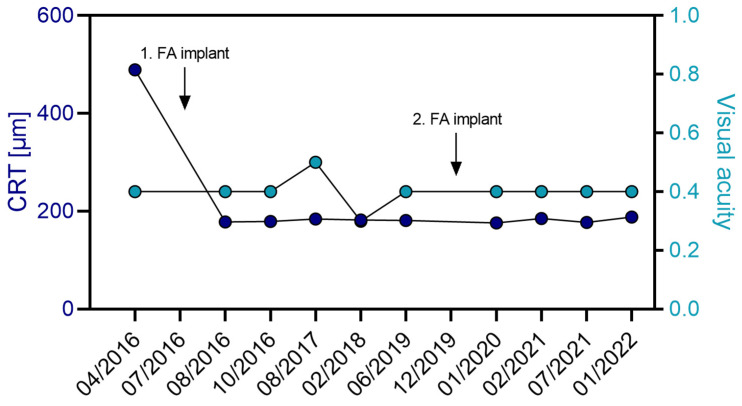
Progression of CRT (µm) and visual acuity in OS of patient A with DME over a follow-up period of 5.75 years. The patient received her first FA implant in July 2016 and the second FA implant 35 months later in December 2019. No additional treatments were necessary for the management of the macular edema. Arrows indicate time point of FA implant injection.

On 12 July 2016, the patient received her first FA implant. CRT decreased from 489 µm to 178 µm within one-month post-FA treatment, remaining consistently below 200 µm for 41 months (Table 3 and Figure 3). After a second FA implant on 19 December 2019, the retina remained dry and below 200 µm for another 25 months. Overall, VA was maintained throughout the entire follow-up. IOP was effectively controlled with IOP-lowering eye drops (brinzolamide), staying below 20 mmHg for the 68-month follow-up period (Table S5 in File S1).

#### 3.6.2. Patient B: Birdshot Retinopathy (OS and OD)

A female 60-year-old patient was HLA-A29-positive and diagnosed with bilateral Birdshot retinopathy. In 2014, she underwent phacoemulsification and intraocular lens implantation in both eyes. Prior to receiving FA implants in both eyes, she was treated with four dexamethasone intravitreal implants in the OD (last in August 2013) and four in the OS (last in September 2013).

**Figure 4:**
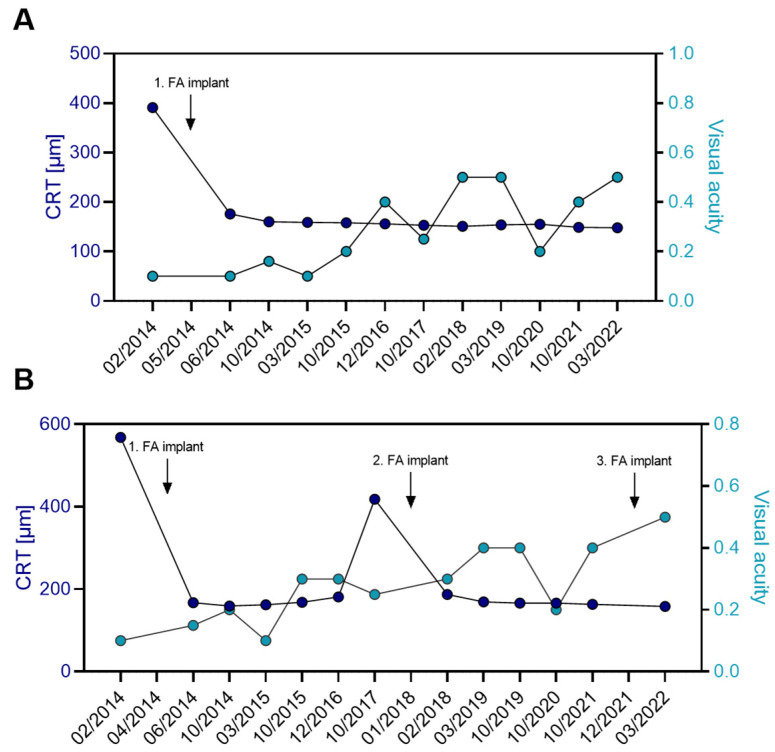
Progression of CRT (µm) and visual acuity of patient B with Birdshot retinopathy bilaterally treated over a 8-year follow-up period. (**A**) In the patient’s OS, a single FA implant was sufficient to maintain the CRT in constant remission over a follow-up period of nine years. No additional treatments were necessary for controlling inflammation in the left eye. (**B**) In the right eye, a total of three FA implants were administered. The first implant was injected in April 2014, the second implant 45 months later in January 2018, and the third FA implant 47 months later in December 2021. During the 8-year follow-up period, no additional intravitreal or systemic medications were administered to the patient. Arrows indicate time point of FA implant injection.

In the left eye of patient B, the FA implant was administered on 15 May 2014. Uveitic macular edema decreased from 391 µm to 170 µm after one month of FA treatment and remained below 200 µm throughout the 9-year follow-up (Table 4, Figure 4A). The edema went into constant remission with a single FA implant, without the need for additional intravitreal or systemic treatments. VA improved from 0.1 pre-FA treatment and was maintained at 94 months of FA treatment.

The patient received the first FA implant in the right eye on 10 April 2014. Retinal thickness was decreased from 568 µm pre-FA treatment to 189 µm two months post-FA treatment (Table 4, Figure 4B). VA improved from 0.1 baseline to 0.3, 18 months after FA treatment. This effect was maintained until a recurrence occurred 42 months post-FA implantation. Subsequently, the patient received a second FA injection 45 months after the first FA implant. The edema decreased from 418 µm at recurrence to 187 µm one month after the injection, with stable VA. After 46 months, the patient received a third FA implant in the right eye. The macula remained dry (<200 µm) at the three-month follow-up visit, and BCVA improved to 0.5. No additional therapies were administered to the right eye.

The patient’s IOP in the right eye increased from 14 mmHg pre-FA implantation to peaks of 22 mmHg at 11 months and 24 mmHg at 32 months post-FA treatment (Table S6 in File S1). The pressure increase was well-controlled with IOP-lowering eye drops (brinzolamide). No IOP-lowering medications were needed in the left eye (Table S6 in File S1). IOP briefly increased to 20 mmHg at 10 months but remained within the normal range (<21 mmHg) throughout the entire follow-up.

## 4. Discussion

In our retrospective study, we found the FA implant to rapidly reduce and maintain CRT during a follow-up period of up to 36 months in eyes with DME or NIU-PS. While short-acting therapies have also shown success in treating patients, continuous monitoring is needed to identify the point retreatment is needed to avoid the recurrence of edema and fluctuations in CRT. Over time, these fluctuations can rearrange and mechanically damage the retinal layers, leading to vision loss if treatment remains insufficient for a prolonged period. Fluctuations and volume changes in the macula are increasingly discussed as initial studies show a clear negative link between these fluctuations and visual outcomes, regardless of the therapy type [15,16,29,31,33,34,35,36]. For FA, Riemann et al. showed that during FA treatment, CRT fluctuations were significantly reduced, leading to better visual outcomes [15]. Detailed single-case studies also confirm that the switch to FA significantly reduces the fluctuations in CRT compared to treatment with anti-VEGFs and short-acting steroids [37,38]. Our results support these aforementioned findings. We also observed in our cohorts that the FA implant led to a significant reduction in CRT, with an amplitude of 271 µm (DME) and 185 µm (NIU-PS) in the first quartile. These reductions were maintained throughout the follow-up period, resulting in fewer fluctuations and a stabilization of CRT. The detailed case studies of patients with DME (A) or uveitis (B) also show a sustained and long-term reduction in macular thickness without fluctuations over a follow-up period of three years.

Correlations between VA and CRT could not be calculated in this study due to the limited number of eyes. However, our analysis showed an overall stabilization of BCVA during the follow-up period. In the NIU-PS cohort, an improvement in BCVA was observed within the first two years. Especially in patients who have undergone prolonged pre-treatment with various therapies before transitioning to FA and who already exhibit irreversible retinal damage, maintaining VA can be regarded as therapeutic success. Long-term undertreatment, combined with recurring macular edema and repeated fluctuations during treatment with short-acting anti-VEGFs and steroids, leads to mechanical damage in the retinal layers. Once a certain level of damage is reached, VA can no longer recover, and the treatment goal shifts to maintaining stable VA [39]. Patients in our cohorts frequently present with an extensive history of prior treatments. Typically, they are managed with short-acting therapies by local conservative ophthalmologists until macular edema has progressed, VA can no longer recover, and recurrences have become more pronounced. At this point, referrals are made to specialized centers and over-regional facilities, such as the University Hospital of Erlangen. Consequently, cohorts at clinics often represent a collection of patients with worse disease progression. While the type and number of treatments administered at the clinic since patient admission are well-documented, medical records prior to referral to a specialized center often contain gaps. Consequently, the true extent of prior treatments and the duration of macular edema are likely underrepresented in our data. An early switch to FA is well-documented to be crucial for better outcomes for the patients: Khoramnia et al. showed that patients with a shorter duration of DME (≤3.6 years) significantly benefited from an early switch to FA regarding VA and safety parameters compared to patients with DME longer than 3.6 years [21]. It has also been demonstrated that a direct or indirect switch (via dexamethasone implant) to FA after suboptimal anti-VEGF response leads to comparable functional and anatomical outcomes. For patients who do not respond to first-line therapies, a direct switch to FA may avoid the need for repeated clinical visits and improve outcomes [40]. Furthermore, the publication by Abu Arif et al. demonstrated that patients with a higher number of dexamethasone pre-treatments show poorer outcomes following FA implantation. This highlights the potential for better results if patients were switched earlier to long-term corticosteroid therapy with FA [41]. Also, other studies support the benefits of an early switch [40,42].

The patient case of DME presented in detail here (patient A) demonstrates that FA therapy effectively reduces DME (<300 µm) and achieves visual stabilization. This case demonstrates long-term CRT stabilization at a low level, without fluctuations, typically observed with continuous retreatment using short-acting medications. In this patient, no additional treatments were required. However, in cases of severe inflammatory activity, an FA implant alone may sometimes be insufficient to keep the macula dry over a three-year period. In such cases, additional treatments with intravitreal dexamethasone or anti-VEGF injections may be necessary [21,26]. In case of severe baseline inflammatory activity, a remission induction therapy with high-dose steroids prior to FA treatment can help to reduce acute inflammation, whereas a subsequently administered FA implant might achieve effective maintenance of the effects and reduce recurrences [23,28].

The case of patient B with Birdshot retinopathy who received bilateral FA treatment showed that, in one eye, a single FA implant could achieve sustained remission of uveitic macular edema, while in the other eye, three FA injections over a nearly 10-year follow-up period were administered to control inflammation and maintain a dry macula. This case illustrates that reinjection with three implants poses no risk to the patient and is associated with significant functional and anatomical improvements. VA in the patient’s OD improved consistently over the years, with no retinal fluctuations observed, due to the prolonged delivery of the FA implant and the avoidance of recurring short-term intravitreal treatments. In contrast, the administration of a single FA implant was sufficient for maintaining sustained remission of the uveitic inflammation in her left eye. This finding is consistent with observations reported by Hikal et al., who documented a case where the macula remained dry without adjunctive therapy for 60 months following FA implantation [26].

Overall, adverse events occurred in only a small proportion of cases. The percentage of eyes that needed IOP-lowering medication (42.9% in eyes with DME, 32.0% in eyes with NIU-PS) in our study was comparable to or even lower than reported in previous clinical trials or other real-world studies [21,22,27]. Two eyes with NIU-PS developed secondary glaucoma post-FA treatment. For these two eyes, IOP elevations were well-managed with IOP-lowering medication, and no glaucoma was diagnosed before FA administration, hence, there was no contraindication given and patients were treated with FA and included in our analysis. The rate of cataract formation and surgery was reported in 40% (5/21) of eyes with DME and 36% (9/25) of eyes with NIU-PS and was also lower compared to previously published clinical trials [22,25]. Hence, our data show that FA has a good safety profile in our hands in a real-world setting. In general, there is a risk of secondary infections, such as herpes, which is relatively common occurring during immunosuppressive treatment of non-infectious uveitis [43]. However, we did not observe this in our cohort. After FA implantation, treatment frequency could be reduced, in line with low rates of supplementary therapy (9/21 in DME, 5/25 in NIU-PS; see Table 2) and a decrease in CRT fluctuations, which is also comparable to other studies [23,25,34]. This reduced treatment burden is also important for good compliance, whereas in short-acting treatment regimens, follow-up appointments are often missed [44]. Interestingly, the FA implant was reinjected in three eyes of a total number of forty-six eyes, considering that continuation of treatment and follow-up care are often relocated to previous conservative physicians. FA was administered bilaterally in 16.6% (3/18) of patients with DME and 50.0% (9/18) of patients with NIU-PS.

This study’s retrospective nature led to several limitations. The partially incomplete documentation of the number and type of pre-FA treatments, the duration of illness, and the follow-up period do not allow for subgroup analyses. Hence, the cohorts are heterogeneous, and patients vary in the severity of their retinopathy and their responsiveness to the FA implant. Due to the small sample size and the heterogeneity of the patient groups, the statistical power and generalizability of our results are limited.

In summary, in our retrospective real-world study including patients with DME and NIU-PS, we observed sustained structural improvements in combination with improvement and stabilization of VA that could be achieved up to three years with one single intravitreal injection of FA in our cohorts. Fluctuations in CRT were reduced with a tolerable safety profile. The exploratory nature of our findings indicates that the stabilization of CRT fluctuations—combined with a reduced treatment burden—could offer potential benefits of an earlier switch to FA, possibly improving patient compliance and reducing the risk of future retinal damage, ultimately leading to better VA outcomes.

## Figures and Tables

**Table 1:** Baseline demographics of patients with DME and NIU-PS.

Baseline Demographics	DME	NIU-PS
Number of eyes	21	25
Number of patients	18	18
Mean age ± SD (years)	66.6 ± 5.9	55.0 ± 11.4
Sex (*n* (%))		
Female	9 (42.9)	21 (84.0)
Male	12 (57.1)	4 (16.0)
Mean BCVA ± SD	0.25 ± 0.17	0.34 ± 0.26
Mean IOP ± SD (mmHg)	14.5 ± 3.6	13.8 ± 2.8
Lens status (*n* (%))		
Phakic	5 (23.8)	9 (36.0)
Pseudophakic	16 (76.2)	16 (64.0)
Aphakic	0 (0.0)	0 (0.0)
Previous treatment (intravitreal or intraocular) (*n* (%))		
Anti-VEGF only	5 (23.8)	0 (0.0)
Anti-VEGF and steroids	9 (42.9)	1 (4.0)
Anti-VEGF and laser	2 (9.5)	0 (0.0)
Anti-VEGF, laser, and steroids	2 (9.5)	0 (0.0)
Steroids only	2 (9.5)	21 (84.0)
Unknown or no previous treatment	1 (4.8)	2/1 (12.0)
Non-infectious uveitis with involvement of posterior segment (*n* (%))		
Anterior	/	0 (0.0)
Intermediate	/	2 (8.0)
Posterior	/	21 (84.0)
Panuveitis	/	2 (8.0)

**Table 2:** Supplementary intravitreal treatments within 36 months of follow-up in eyes with DME and NIU-PS.

Supplementary Intravitreal Treatments within 36 Months	DME (*n* = 21)	NIU-PS (*n* = 25)
Eyes with supplementary treatments (*n* (%))	9 (42.9)	5 (20.0)
Type of supplementary treatments (*n* eyes (% of total eyes with supplementary treatments))		
Intravitreal steroids	5 (55.6)	2 (40.0)
Intravitreal anti-VEGF	7 (77.8)	4 (16.0)
Laser therapy	3 (33.3)	0 (0.0)
Combination of different treatments	3 (33.3)	1 (20.0)
Number of treatments (mean ± SD)	4.6 ± 4.1	4.8 ± 5.4

**Table 3:** Visual acuity, CRT data, and OCT images for patient A with DME (OS).

Date	Visual Acuity	CRT (µm)	OCT	Treatment
April 2016	0.4	489	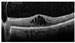	1. FA implant (07/2016)
August 2016	0.4	178	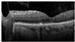	
October 2016	0.4	179	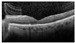	
August 2017	0.5	184	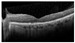	
February 2018	0.3	182	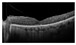	
June 2019	0.4	181	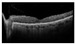	2. FA implant (12/2019)
January 2020	0.4	176	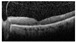	
February 2021	0.4	185	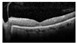	
July 2021	0.4	177	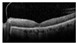	
January 2022	0.4	188	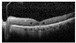	

**Table 4:** Visual acuity, CRT data, and OCT images for patient B with Birdshot retinopathy (OD and OS).

	OS				OD			
Date	Visual Acuity	CRT (µm)	OCT	Treatment	Visual Acuity	CRT (µm)	OCT	Treatment
February 2014	0.1	391	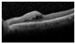	1. FA implant (05/2014)	0.1	568	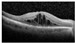	1. FA implant (04/2014)
June 2014	0.1	176	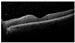		0.15	167	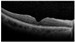	
October 2014	0.16	160	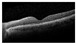		0.2	159	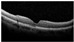	
March 2015	0.1	159	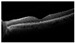		0.1	162	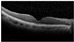	
October 2015	0.2	158	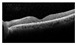		0.3	168	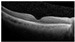	
December 2016	0.4	156	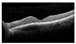		0.3	181	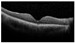	
October 2017	0.25	153	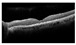		0.25	418	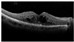	2. FA implant (01/2018)
February 2018	0.5	151	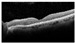		0.3	187	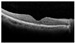	
March 2019	0.5	154	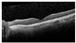		0.4	169	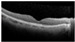	
October 2019	/	/	/		0.4	166	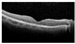	
October 2020	0.2	155	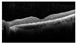		0.2	166	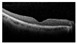	
October 2021	0.4	149	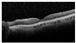		0.4	163	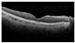	3. FA implant (12/2021)
March 2022	0.5	148	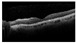		0.5	158	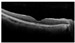	

## Data Availability

The data presented in this study are available on request from the corresponding author due to privacy and ethical reasons.

## Supplementary Materials

The following supporting information can be downloaded at https://www.mdpi.com/article/10.3390/jcm14082849/s1, Supporting information File S1: Pre-treatments, IOP-related events, and IOP progression of patient cases.

## Abbreviations

The following abbreviations are used in this manuscript:

BCVA	Best-corrected visual acuity
CME	Chronic macular edema
CRT	Central retinal thickness
DME	Diabetic macular edema
FA	Fluocinolone acetonide
IOP	Intraocular pressure
NIU-PS	Non-infectious uveitis affecting the posterior segment
OCT	Optical coherence tomography
OD	Oculus dexter
OS	Oculus sinister
SD	Standard deviation
SEM	Standard error mean
TNF	Tumor necrosis factor
VA	Visual acuity
VEGF	Vascular endothelial growth factor

## References

[1] Magliano D.J., Boyko E.J. (2021). IDF Diabetes Atlas.

[2] Kraatz K., Neu A., Kröger J., Seidel-Jacobs E., Tönnies T., Rathmann W., Schulze M., Kabisch S., Sachno A., Ramich O. (2023). Deutscher Gesundheitsreport Diabetes.

[3] Ong K.L., Stafford L.K., McLaughlin S.A., Boyko E.J., Vollset S.E., Smith A.E., Dalton B.E., Duprey J., Cruz J.A., Hagins H. (2023). Global, Regional, and National Burden of Diabetes from 1990 to 2021, with Projections of Prevalence to 2050: A Systematic Analysis for the Global Burden of Disease Study 2021. Lancet.

[4] Dong N., Xu B., Wang B., Chu L. (2013). Study of 27 Aqueous Humor Cytokines in Patients with Type 2 Diabetes with or without Retinopathy. Mol. Vis..

[5] Noma H., Yasuda K., Shimura M. (2021). Involvement of Cytokines in the Pathogenesis of Diabetic Macular Edema. Int. J. Mol. Sci..

[6] Durrani O.M., Meads C.A., Murray P.I. (2004). Uveitis: A Potentially Blinding Disease. Ophthalmologica.

[7] Rosenbaum J.T., Bodaghi B., Couto C., Zierhut M., Acharya N., Pavesio C., Tay-Kearney M.L., Neri P., Douglas K., Pathai S. (2019). New Observations and Emerging Ideas in Diagnosis and Management of Non-Infectious Uveitis: A Review. Semin. Arthritis Rheum..

[8] Tsirouki T., Dastiridou A., Symeonidis C., Tounakaki O., Brazitikou I., Kalogeropoulos C., Androudi S. (2018). A Focus on the Epidemiology of Uveitis. Ocul. Immunol. Inflamm..

[9] Pleyer U., Neri P., Deuter C. (2021). New Pharmacotherapy Options for Noninfectious Posterior Uveitis. Int. Ophthalmol..

[10] Sivaprasad S., Oyetunde S. (2016). Impact of Injection Therapy on Retinal Patients with Diabetic Macular Edema or Retinal Vein Occlusion. Clin. Ophthalmol..

[11] Ehlken C., Ziemssen F., Eter N., Lanzl I., Kaymak H., Lommatzsch A., Schuster A.K. (2020). Systematic Review: Non-Adherence and Non-Persistence in Intravitreal Treatment. Graefe’s Arch. Clin. Exp. Ophthalmol..

[12] Weiss M., Sim D.A., Herold T., Schumann R.G., Liegl R., Kern C., Kreutzer T., Schiefelbein J., Rottmann M., Priglinger S. (2018). Complicance and Adherence of Patients with Diabetic Macular Edema to Intravitreal Anti-Vascular Endothelial Growth Factor Therapy in Daily Practice. Retina.

[13] Daruich A., Matet A., Moulin A., Kowalczuk L., Nicolas M., Sellam A., Rothschild P.R., Omri S., Gélizé E., Jonet L. (2018). Mechanisms of Macular Edema: Beyond the Surface. Prog. Retin. Eye Res..

[14] Perez V.L., Caspi R.R. (2015). Immune Mechanisms in Inflammatory and Degenerative Eye Disease. Trends Immunol..

[15] Riemann C.D., Eaton A.M., Cutino A. (2020). Reduction in Retinal Thickness Fluctuations after Treatment with Fluocinolone Acetonide Implant for DME: A Post-Hoc Analysis of the User Study. Ophthalmic Surg. Lasers Imaging Retin..

[16] Starr M.R., Salabati M., Mahmoudzadeh R., Patel L.G., Ammar M.J., Hsu J., Garg S., Ho A.C., Kuriyan A.E. (2021). Fluctuations in Central Subfield Thickness Associated With Worse Visual Outcomes in Patients With Diabetic Macular Edema in Clinical Trial Setting. Am. J. Ophthalmol..

[17] Torjani A., Mahmoudzadeh R., Salabati M., Cai L., Hsu J., Garg S., Ho A.C., Yonekawa Y., Kuriyan A.E., Starr M.R. (2023). Factors Associated with Fluctuations in Central Subfield Thickness in Patients with Diabetic Macular Edema Using Diabetic Retinopathy Clinical Research Protocols T and V. Ophthalmol. Sci..

[18] Campochiaro P.A., Nguyen Q.D., Hafiz G., Bloom S., Brown D.M., Busquets M., Ciulla T., Feiner L., Sabates N., Billman K. (2013). Aqueous Levels of Fluocinolone Acetonide after Administration of Fluocinolone Acetonide Inserts or Fluocinolone Acetonide Implants. Ophthalmology.

[19] Deuchler S.K., Schubert R., Singh P., Chedid A., Kenikstul N., Scott J., Kohnen T., Ackermann H., Koch F. (2022). Vitreous Cytokine Levels Following the Administration of a Single 0.19 Mg Fluocinolone Acetonide (ILUVIEN^®^) Implant in Patients with Refractory Diabetic Macular Edema (DME)—Results from the ILUVIT Study. Graefe’s Arch. Clin. Exp. Ophthalmol..

[20] Wykoff C.C., Chakravarthy U., Campochiaro P.A., Bailey C., Green K., Cunha-Vaz J. (2017). Long-Term Effects of Intravitreal 0.19 Mg Fluocinolone Acetonide Implant on Progression and Regression of Diabetic Retinopathy. Ophthalmology.

[21] Khoramnia R., Peto T., Koch F., Taylor S.R., Castro De Sousa J.P., Hill L., Bailey C., Chakravarthy U. (2022). Clinical Science Safety and Effectiveness of the Fluocinolone Acetonide Intravitreal Implant (ILUVIEN): 3-Year Results from the European IRISS Registry Study. Br. J. Ophthalmol..

[22] Campochiaro P.A., Brown D.M., Pearson A., Chen S., Boyer D., Ruiz-Moreno J., Garretson B., Gupta A., Hariprasad S.M., Bailey C. (2012). Sustained Delivery Fluocinolone Acetonide Vitreous Inserts Provide Benefit for at Least 3 Years in Patients with Diabetic Macular Edema. Ophthalmology.

[23] Buhl L., Thurau S., Kern C. (2022). Fluocinolone Acetonide 0.19-Mg Implant for the Treatment of Noninfectious Uveitis with Involvement of the Posterior Segment: A Real-World Study. Graefe’s Arch. Clin. Exp. Ophthalmol..

[24] Jaffe G.J., Pavesio C.E. (2020). Effect of a Fluocinolone Acetonide Insert on Recurrence Rates in Noninfectious Intermediate, Posterior, or Panuveitis: Three-Year Results. Ophthalmology.

[25] Pavesio C., Heinz C. (2021). Non-Infectious Uveitis Affecting the Posterior Segment Treated with Fluocinolone Acetonide Intravitreal Implant: 3-Years Fellow Eye Analysis. Eye.

[26] Hikal M., Celik N., Auffarth G.U., Khoramnia R., Kessler L.J., Mayer C.S. (2021). Intravitreal 0.19 Mg Fluocinolone Acetonide Implant in Non-Infectious Uveitis. J. Clin. Med..

[27] Augustin A.J., Bopp S., Fechner M., Holz F., Sandner D., Winkgen A.M., Khoramnia R., Neuhann T., Warscher M., Spitzer M. (2020). Three-Year Results from the Retro-IDEAL Study: Real-World Data from Diabetic Macular Edema (DME) Patients Treated with ILUVIEN^®^ (0.19 Mg Fluocinolone Acetonide Implant). Eur. J. Ophthalmol..

[28] Buhl L., Schmelter V., Schworm B., Thurau S., Kern C. (2023). Long-Term Results of 0.19mg Fluocinolone Acetonide Insert for Treatment of Non-Infectious Uveitis in Clinical Practice. Ocul. Immunol. Inflamm..

[29] Schechet S.A., Adams O.E., Eichenbaum D.A., Hariprasad S.M. (2019). Macular Thickness Amplitude Changes When Switching from Discontinuous to Continuous Therapy for Diabetic Macular Oedema. BMJ Open Ophthalmol..

[30] Holden S.E., Habib M., Currie C.J. (2020). Retinal Thickness Fluctuations in Patients Receiving Fluocinolone Acetonide Implant for Diabetic Macular Edema. Curr. Med. Res. Opin..

[31] Singer M.A., Sheth V., Mansour S.E., Coughlin B., Gonzalez V.H. (2022). Three-Year Safety and Efficacy of the 0.19-Mg Fluocinolone Acetonide Intravitreal Implant for Diabetic Macular Edema: The PALADIN Study. Ophthalmology.

[32] Sheth V.S., Singer M., MacCumber M., Cutino A., Kasper J., Coughlin B.A., Riemann C.D. (2023). Long-Term Control of Retinal Thickness Variability and Vision Following the 0.19 Mg Fluocinolone Acetonide Implant. J. Vitr. Dis..

[33] Browning D.J., Glassman A.R., Aiello L.P., Beck R.W., Brown D.M., Fong D.S., Bressler N.M., Danis R.P., Kinyoun J.L., Diabetic Retinopathy Clinical Research Network (2007). Relationship between Optical Coherence Tomography-Measured Central Retinal Thickness and Visual Acuity in Diabetic Macular Edema. Ophthalmology.

[34] Kodjikian L., Baillif S., Creuzot-Garcher C., Delyfer M.N., Matonti F., Weber M., Mathis T. (2021). Real-World Efficacy and Safety of Fluocinolone Acetonide Implant for Diabetic Macular Edema: A Systematic Review. Pharmaceutics.

[35] Tomkins-Netzer O., Lightman S.L., Burke A.E., Sugar E.A., Lim L.L., Jaffe G.J., Altaweel M.M., Kempen J.H., Holbrook J.T., Jabs D.A. (2021). Seven-Year Outcomes of Uveitic Macular Edema: The Multicenter Uveitis Steroid Treatment Trial and Follow-up Study Results. Ophthalmology.

[36] Wang V.Y., Kuo B.L., Chen A.X., Wang K., Greenlee T.E., Conti T.F., Singh R.P. (2022). Fluctuations in Macular Thickness in Patients with Diabetic Macular Oedema Treated with Anti-Vascular Endothelial Growth Factor Agents. Eye.

[37] Singh P., Chedid A., Deuchler S.K., Kohnen T., Müller M., Koch F.H. (2018). The Efficacy and Safety Outcomes of the 0.19 Mg Fluocinolone Acetonide Implant after Prior Treatment with the 0.7 Mg Dexamethasone Implant in Patients with Diabetic Macular Edema. Int. Med. Case Rep. J..

[38] McGregor F., Dick A.D., Burke T. (2021). Achieving Quiescence with Fluocinolone Implants. Case Rep. Ophthalmol..

[39] Bodaghi B., Nguyen Q.D., Jaffe G., Khoramnia R., Pavesio C. (2020). Preventing Relapse in Non-Infectious Uveitis Affecting the Posterior Segment of the Eye—Evaluating the 0.2 Μg/Day Fluocinolone Acetonide Intravitreal Implant (ILUVIEN^®^). J. Ophthalmic. Inflamm. Infect..

[40] Rehak M., Busch C., Unterlauft J.D., Jochmann C., Wiedemann P. (2020). Outcomes in Diabetic Macular Edema Switched Directly or after a Dexamethasone Implant to a Fluocinolone Acetonide Intravitreal Implant Following Anti-VEGF Treatment. Acta Diabetol..

[41] Abu Arif J., Knecht V.A., Rübsam A., Lussac V., Jami Z., Pohlmann D., Müller B., Pleyer U. (2024). Fluocinolone Acetonide Implant for Uveitis: Dissecting Responder and Non-Responder Outcomes at a Tertiary Center. Biomedicines.

[42] Eaton A., Koh S.S., Jimenez J., Riemann C.D. (2019). The USER Study: A Chart Review of Patients Receiving a 0.2 Lg/Day Fluocinolone Acetonide Implant for Diabetic Macular Edema. Ophthalmol. Ther..

[43] Wu X., Tao M., Zhu L., Zhang T., Zhang M. (2023). Pathogenesis and Current Therapies for Non-Infectious Uveitis. Clin. Exp. Med..

[44] Glassman A.R., Wells J.A., Josic K., Maguire M.G., Antoszyk A.N., Baker C., Beaulieu W.T., Elman M.J., Jampol L.M., Sun J.K. (2020). Five-Year Outcomes after Initial Aflibercept, Bevacizumab, or Ranibizumab Treatment for Diabetic Macular Edema (Protocol T Extension Study). Ophthalmology.

