# Supplementary material for: Stabilizing Macular Edema Fluctuations: Outcomes of Intravitreal Fluocinolone Acetonide for Diabetic Macular Edema and Non-Infectious Uveitis
Source: J Clin Med. 2025 Apr 21;14(8):2849. doi: 10.3390/jcm14082849 (PMC12027787; doi:10.3390/jcm14082849)
Supplement: Supplementary file 1 [file jcm-14-02849-s001.zip › jcm-3549400-supplementary.pdf]

Supporting Information S1

Table S1. Intravitreal steroid and anti-VEGF treatments prior to FA implantation in DME patients.

**Anti-VEGF and steroid treatments prior to FA implantation in DME patients**	
**Number of eyes**	21
**Steroids (n (%))**	14 (66.7)
**Dexamethasone (n (%))**	
Dexamethasone implants	13 (61.9)
Maximal two dexamethasone implants	8 (38.1)
**Triamcinolone (n (%))**	
Triamcinolone injections	4 (19.0)
Maximal two triamcinolone injections	1 (4.8)
**Anti-VEGF (n (%))**	17 (81.0)
**Laser (n (%))**	3 (14.3)

Table S2. Intravitreal steroid and anti-VEGF treatments prior to FA implantation in NIU-PS patients.

**Anti-VEGF and steroid treatments prior to FA implantation in NIU-PS patients**	
**Number of eyes**	25
**Steroids (n (%))**	22 (88.0)
**Dexamethasone (n (%))**	
Dexamethasone implants	22 (88.0)
Maximal two dexamethasone implants	13 (52.0)
**Triamcinolone (n (%))**	1 (4.0)
**Anti-VEGF (n (%))**	1 (4.0)
**Laser (n (%))**	0 (0.0)

Table S3. IOP-related events in DME eyes.

Occurrence of IOP-related events in DME eyes	
**IOP-related events**	**All DME eyes (n=21)**
IOP pre-FA (mmHg ± SD)	14.5 ± 3.6
IOP increase of ≥ 10 mmHg (n (% eyes))	3 (14.3)
IOP 21-30 mmHg during follow-up (n (% eyes))	3 (14.3)
IOP ≥ 30 mmHg during follow-up (n (% eyes))	4 (19.0)
IOP lowering medication pre-FA (n (% eyes))	2 known (9.5)
IOP lowering medication during follow-up (n (% eyes))	9 (42.9)
IOP lowering surgery (n (% eyes))	0 (0.0)

Table S4. IOP-related events in NIU-PS eyes.

Occurrence of IOP-related events in NIU-PS eyes	
**IOP-related events**	**All NIU-PS eyes (n=25)**
IOP pre-FA (mmHg ± SD)	13.8 ± 2.8
IOP increase of ≥ 10 mmHg (n (% eyes))	6 (24.0)
IOP 21-30 mmHg during follow-up (n (% eyes))	7 (33.3)
IOP ≥ 30 mmHg during follow-up (n (% eyes))	4 (16.0)
IOP lowering medication pre-FA (n (% eyes))	2 known (7.7)
IOP lowering medication during follow-up (n (% eyes))	8 (32.0)
IOP lowering surgery (n (% eyes))	3 (12.0)

Table S5. IOP progression of DME patient A before and after FA implantation.

**IOP progression of DME patient A**	
**Date**	**IOP [mmHg]**
04/2016	12
07/2016	1. FA implant
08/2016	18
01/2017	12
04/2017	12
08/2017	16
01/2018	16
04/2018	11
07/2018	14
12/2018	16
06/2019	16
12/2019	2. FA implant
01/2020	14
02/2021	16
07/2021	14
01/2022	12

Table S6. IOP progression of bilaterally treated Birdshot patient B before and after FA implantation.

**IOP progression of Birdshot patient B (OD & OS)**		
**Date**	**IOP [mmHg] (OD)**	**IOP [mmHg] (OS)**
02/2014	14	14
04/2014	1. FA implant	/
05/2014	/	1. FA implant
06/2014	16	18
10/2014	14	14
03/2015	22	20
06/2015	12	12
10/2015	16	15
12/2016	24	19
03/2017	13	12
06/2017	11	13
10/2017	11	12
01/2018	2. FA implant	/
02/2018	10	10
10/2018	18	18
03/2019	19	18
10/2019	18	17
10/2019	18	16
03/2021	13	14

10/2021	13	14
12/2021	3. FA implant	/
03/2022	15	15
